# Role of *Drosophila* in Human Disease Research 3.0

**DOI:** 10.3390/ijms25010292

**Published:** 2023-12-25

**Authors:** Sue Cotterill, Masamitsu Yamaguchi

**Affiliations:** 1 Molecular and Clinical Sciences Research Institute, St George’s University of London, London SW17 0RE, UK; 2 Kansai Gakken Laboratory, Kankyo Eisei Yakuhin Co., Ltd., 3-6-2 Hikaridai, Seika-cho, Kyoto 619-0237, Japan; myamaguc8@gmail.com

*Drosophila melanogaster* has become a commonly used animal model for biomedical research in a variety of areas. About 85% of human disease-related genes have *Drosophila* homologs, and the most important biological pathways are well conserved between these two species [1]. This suggests that the results obtained in relation to *Drosophila* will be applicable to mammalian systems. In addition, *Drosophila* has a number of advantages that facilitate rapid progress. *Drosophila* has a relatively short life span, allowing for the rapid acquisition of experimental data. *Drosophila* produces a large number of progeny, facilitating statistical analyses of the obtained data. In addition, experiments with *Drosophila* can be performed with fewer ethical concerns than those with rodent models. More importantly, many genetic tools are available in terms of *Drosophila*, including well-established genetic techniques and a large number of mutants and transgenic lines for overexpression or knockdown that can easily be obtained from several stock centers that exist worldwide. Thus, various *Drosophila* models have been developed, and they are commonly used for the elucidation of the in vivo functions of human disease-related genes and the identification of candidate substances for therapies concerning corresponding human diseases.

This is the third Special Issue focusing on this subject. Volume 1 included 12 articles (https://www.mdpi.com/journal/ijms/special_issues/Drosophila_Human_Diseases (accessed on 6 November 2023)) that covered a wide variety of human neuropathies, including Alzheimer’s disease (AD), epileptic encephalopathy (EE), autism spectrum disorders (ASD) and some other rare diseases and disorders. Volume 2 included nine articles (https://www.mdpi.com/journal/ijms/special_issues/Drosophila_Human_Diseases_2 (accessed on 6 November 2023)) that covered other human diseases and disorders, such as oral cancer in relation to noncoding microRNA (miRNA), obesity, diabetes, Parkinson’s disease (PD), amyotrophic lateral sclerosis (ALS), Charcot–Marie–Tooth disease (CMT) and infectious diseases. As summarized below, the third volume of this Special Issue (https://www.mdpi.com/journal/ijms/special_issues/Drosophila_Human_Diseases_3 (accessed on 6 November 2023)) includes 10 articles that further expand the discussion to other human diseases and disorders, such as those related to immune system and replicative DNA polymerase gene mutations. It also further deepened the previously covered topics, such as neurodegenerative diseases, alcohol use disorder and communicable diseases.

Two research articles published in this volume focused on the utilization of *Drosophila* when studying neurodegenerative diseases. In the first article, Santos-Cruz et al. used *Drosophila* to study the metabolic effects caused by the consumption of fructose and palmitic acid [2]. It is known that highly energetic ultra-processed foods contain fructose and palmitic acid, which appear to play a role in the progression of various diseases and disorders, including cardiovascular and neurodegenerative diseases. Transcriptomic profiling in the brain and midgut of *Drosophila* larvae fed a diet supplemented with fructose and palmitic acid revealed that the biosynthesis of proteins at the mRNA level that participate in the synthesis of amino acids and enzymes for the dopaminergic and GABAergic systems are altered in these organs [2]. Their findings may provide a deeper understanding of the mechanisms through which the consumption of these alimentary products is related to the development of neuronal diseases. In the second paper, by using *Drosophila*, Lee et al. demonstrated that ZnT86D, a *Drosophila* ortholog of zinc transporter ZnT7, plays an important role in the neurodevelopment and pathogenesis of AD [3]. RNAi silencing of *ZnT86D* in neurons induced abnormal neurogenesis and neuronal cell death that resulted in reduced survival rates, locomotor activity, and lifespan. Interestingly, neuron-specific knockdown of *ZnT86D* in the AD model fly enhanced apoptosis and neurodegeneration without alterations in the deposition of amyloid beta and susceptibility to oxidative stress [3]. Since ZnT7 is mainly localized in the Golgi apparatus and endoplasmic reticulum (ER), their findings suggest that proper distribution of zinc in the Golgi apparatus and ER is critical for neuronal development and neuroprotection.

Two articles focused on immunomodulators. The innate immune system is highly conserved between *Drosophila* and humans, and therefore, *Drosophila* is a useful model when studying the molecular mechanism of the host’s innate immune response. Using *Drosophila*, Won et al. clarified a novel role of NSD in immune responses [4]. NSD is a *Drosophila* ortholog of human NSD1, a histone H3 lysine 36 (H3K36)-specific methyltransferase. The overexpression of NSD in the fat body caused an increase in the mRNA levels of anti-microbial peptides [4]. A more detailed study revealed that transcriptional activation by NSD is mainly mediated through the immune deficiency (IMD) pathway by activating Relish. As expected, the survival rate of the NSD-overexpressing larvae was increased via the oral ingestion of Gram-negative bacteria [4]. Thus, NSD is associated with the IMD pathway. These findings may provide a deeper understanding of the mechanisms of immune malfunction observed in various NSD1-associated human diseases. Plants contain various compounds that function as immunomodulators. In their article, Pratomo et al. emphasized that *Drosophila* is a powerful model organism in terms of identifying such compounds from plant extracts [5]. The authors summarized seminal contributions made by *Drosophila* scientists on this subject [5]. More than 70 examples of the immunomodulatory effects of plant/plant-derived compounds screened by using *Drosophila* are listed in this review. They contain diverse and complex blends of bioactive compounds, such as polyphenols, anthraquinones, and flavonoids, which very likely contribute to the beneficial modulation of the immune system [5].

One article in this volume provided an example of the use of *Drosophila* to examine the effect of a plant-containing diet. Lopez-Ortiz et al. focused on a habanero pepper and used *Drosophila* to examine the molecular and physiological changes associated with dietary pepper consumption [6]. The authors carried out genome-wide transcriptome and metabolome analyses of adult flies fed a habanero pepper diet and showed a list of upregulated or downregulated genes and metabolites [6]. These comprehensive studies of the response to a pepper diet in *Drosophila* may provide fundamental information for a deeper understanding of the molecular mechanism of pepper consumption.

Continuing with a dietary theme, Contreras et al. [7] studied the effects of inositol in relation to disease and development. Inositol is a type of sugar abundant in a variety of tissues that can be obtained from foods such as fruit, beans, grains, and nuts. It has been associated with a number of cellular and metabolic functions, including membrane formation and cellular and neural signaling, and has been suggested as being beneficial in terms of diabetes, PCOS, and some neurological disorders. The study presented here looked specifically at inositol catabolism via myo-inositol oxygenase (MIOX). By manipulating the levels of this enzyme, the authors were able to show that the complete elimination of MIOX caused high levels of myo-inositol, which resulted in developmental defects and lethality. However, a more moderate increase in levels was not harmful and could actually have some positive effects, such as reduced obesity and glucose levels, which might have implications for managing these conditions in human health.

Two papers looked at the interaction between harmful dietary components and metabolism. Alcohol abuse and excess stimulant usage are both important public health problems. De Nobrega et al. [8] discussed the interaction between sleep and alcohol toxicity and showed that sleep deprivation in flies caused increased toxicity without affecting alcohol absorption or clearance. The toxic effects of alcohol could be diminished if sleep was pharmacologically induced prior to alcohol intake. The effects held for both males and females and also in flies with a variety of vulnerabilities. Cummins-Beebee et al. [9] were more concerned with the addiction mechanisms of alcohol and psychostimulants. They reviewed ways in which the tools available in *Drosophila* can be used to study physiological adaptations to tolerance, dependence and withdrawal of these substances and identify the genes that are involved in such responses. They highlighted the conservation of these genes in higher systems and suggested the uses and limitations of this research in its application to mammalian systems.

The final two papers looked at how mutations in fundamental conserved cellular protein families are associated with disease and how research that uses *Drosophila* can help to dissect the mechanisms of these effects. Yamaguchi and Cotterill [10] presented a review of how mutations in a very well-conserved group of proteins, replicative DNA polymerases, cause a variety of human diseases and how studies in *Drosophila* have contributed to a mechanistic understanding of normal and corrupted polymerase functioning. They ended by suggesting ways in which the use of *Drosophila* models could contribute to deepening the understanding of disorders associated with mutations in this group of proteins. Scholl and De [11] focused on another important group, polycomb group proteins (PcG). They discussed the conservation of PcG proteins between *Drosophila* and mammals. They then went on to focus on how this group of proteins functions in relation to the response to various types of pathogenic infections via their effect on epigenetic controls and protein interactions and how the results obtained from studies using *Drosophila* could contribute to the development of therapeutics against these important groups of human pathogens.

In summary, the papers in this Special Issue further the case for the role of *Drosophila* in medical research. They demonstrate that the results gained from *Drosophila* already have applications in terms of understanding a wide range of diseases and show how further analysis using this organism will be able to provide further insight into both the mechanisms of diseases and their treatments.
